# Antiretroviral Therapy Intensification With Dolutegravir and/or Maraviroc Did Not Affect HIV-1 Cell-Associated DNA, RNA, and 2­–LTR Circles Over 12 Weeks

**DOI:** 10.1093/ofid/ofaf594

**Published:** 2025-10-01

**Authors:** Jonathan C Reed, Luke Hall, Ashley McKhann, Ginger Kwak, Erin A Goecker, Robert W Coombs, Huichao Chen, Jhoanna Roa, Alyssa Vecchio, Eric S Daar, Peter W Hunt, Christina M Marra, Thomas B Campbell, Qing Ma, Shobha Swaminathan, Bernard J C Macatangay, Gene D Morse, Thomas Miller, David Rusin, Belinda Ha, Beverly Alston-Smith, Robert Paul, Scott L Letendre, Serena S Spudich, Alexander L Greninger

**Affiliations:** Department of Laboratory Medicine and Pathology, University of Washington School of Medicine, Seattle, Washington, USA; Department of Biostatistics, Harvard T. H. Chan School of Public Health, Boston, Massachusetts, USA; Department of Biostatistics, Harvard T. H. Chan School of Public Health, Boston, Massachusetts, USA; Department of Laboratory Medicine and Pathology, University of Washington School of Medicine, Seattle, Washington, USA; Department of Laboratory Medicine and Pathology, University of Washington School of Medicine, Seattle, Washington, USA; Department of Laboratory Medicine and Pathology, University of Washington School of Medicine, Seattle, Washington, USA; Department of Biostatistics, Harvard T. H. Chan School of Public Health, Boston, Massachusetts, USA; DLH Corporation, Bethesda, Maryland, USA; University of North Carolina, Chapel Hill, North Carolina, USA; Lundquist Institute at Harbor–University of California–Los Angeles Medical Center, Torrance, California, USA; Department of Medicine, University of California–San Francisco, San Francisco, California, USA; Department of Laboratory Medicine and Pathology, University of Washington School of Medicine, Seattle, Washington, USA; Department of Medicine, University of Colorado School of Medicine, Aurora, Colorado, USA; Department of Pharmacy Practice, University at Buffalo, Buffalo, New York, USA; Department of Medicine, Rutgers New Jersey Medical School, Newark, New Jersey, USA; Department of Medicine, University of Pittsburgh, Pittsburgh, Pennsylvania, USA; Department of Pharmacy Practice, University at Buffalo, Buffalo, New York, USA; Frontier Science Foundation, Amherst, New York, USA; Frontier Science Foundation, Amherst, New York, USA; ViiV Healthcare, Research Triangle Park, North Carolina, USA; Division of AIDS, National Institutes of Health, Rockville, Maryland, USA; Department of Psychological Sciences, University of Missouri, St Louis, Missouri, USA; Department of Medicine, University of California–San Diego, San Diego, California, USA; Department of Neurology, Yale School of Medicine, New Haven, Connecticut, USA; Department of Laboratory Medicine and Pathology, University of Washington School of Medicine, Seattle, Washington, USA; Vaccine and Infectious Disease Division, Fred Hutchinson Cancer Research Center, Seattle, Washington, USA

**Keywords:** HIV, reservoir, ART intensification, dolutegravir, maraviroc, 2-LTR, ddPCR

## Abstract

**Background:**

Neurocognitive impairment (NCI) among people living with human immunodeficiency virus (HIV; PWH) on antiretroviral therapy (ART) may result from residual viral replication. The A5324 trial found that ART intensification with dolutegravir (DTG) with or without maraviroc (MVC) did not affect NCI in PWH. We evaluated the impact of ART intensification on peripheral virological measures during the first 12 weeks of intensification.

**Methods:**

The A5324 study was a randomized, double-blind, placebo (PBO)–controlled, 96-week trial of ART intensification with either dual PBO, DTG + PBO, or DTG + MVC in PWH with NCI on ART who were naive to integrase strand transfer inhibitors and MVC. At baseline and weeks 2, 4, and 12, HIV-1 RNA was measured in plasma with a low-copy assay, while HIV-1 cell-associated DNA (caDNA), cell-associated unspliced RNA (caRNA), and cell-associated 2-long terminal repeat circles (ca2LTR) were quantified from peripheral blood mononuclear cells using droplet digital polymerase chain reaction.

**Results:**

Of the 171 participants, 59 were randomized to dual PBO, 57 to DTG + PBO, and 55 to DTG + MVC. Changes in caDNA and caRNA and detection of plasma RNA did not differ between treatment arms over 12 weeks (*P* > 0.05). Detection of ca2LTR was less frequent at weeks 2–4 in the DTG + MVC arm (40.4%) than in the dual-PBO (70.7%; *P* =0 .02) and DTG + PBO (68.4%; *P* = 0.03) arms. However, this difference diminished by week 12, and baseline ca2LTR detection in the DTG + MVC arm was lower than in the other groups.

**Conclusions:**

DTG intensification had no effect on peripheral markers of HIV-1 persistence. DTG + MVC intensification reduced ca2LTR detection at weeks 2–4, though this effect did not persist through week 12. These findings indicate the minimal impact of intensification on the HIV-1 peripheral reservoir, consistent with prior studies.

## Teaser

In people with HIV-1 and with neurocognitive impairment, the A5324 trial tested the effect of antiretroviral therapy (ART) intensification with either dolutegravir or dolutegravir + maraviroc. ART intensification did not significantly affect virological measures of the HIV-1 peripheral reservoir.

Antiretroviral therapy (ART) has been highly effective at controlling human immunodeficiency virus (HIV) viremia, allowing people living with HIV (PWH) to avoid disease progression. PWH still experience higher rates of comorbid condition than people without HIV, including HIV-associated neurocognitive (NC) impairment (NCI). Based on the Frascati criteria for HIV-associated NC disorders (HAND), established in 2007 [1], the prevalence of NCI among PWH generally ranges from 20% to 60% [2]. Of PWH with NCI, most experience asymptomatic NCI, the mildest form, while those with unsuppressed viral loads and lower nadir CD4 cell counts have a greater risk of experiencing either mild NC disorder or HIV-associated dementia [3]. Several studies have attempted to address the possibility of ongoing HIV-1 replication in the central nervous system (CNS) through the use of antiretrovirals with improved distribution into the CNS; however, the results have been mixed, and there is continued controversy regarding this strategy [4–6].

The A5324 study was a randomized, double-blind, placebo (PBO)–controlled clinical trial to evaluate the effects of ART intensification on NCI in PWH [7]. Study participants were virologically suppressed and randomized to continue their current ART plus dual PBO, the integrase strand transfer inhibitor (INSTI) dolutegravir (DTG) with PBO, or the combination of DTG with the CCR5 entry inhibitor maraviroc (MVC). These 2 drugs were selected due to improved CNS distribution [8–10] and the ability of MVC to block the main coreceptor for HIV in CNS-resident macrophages. The A5324 trial found that ART intensification with DTG or DTG + MVC did not improve NCI [7].

Before the A5324 trial, 2 studies reported that intensification with raltegravir, a first-generation INSTI, appeared to suppress residual replication, as evidenced by a transient increase in cell-associated 2–long terminal repeat (LTR) circles (ca2LTR) levels in peripheral blood mononuclear cells (PBMCs) within the first 2–4 weeks of intensification [11, 12]. This finding was controversial, as other raltegravir intensification studies did not observe impacts on ca2LTR levels, but those studies did not include early sampling [13, 14]. One of the exploratory aims of the A5324 trial was to determine whether intensification with the improved second-generation INSTI DTG might also be associated with early transient increases in ca2LTR. To match the design of the earlier studies that detected this change, A5324 included sampling early after intensification (weeks 2 and 4) and measurements of ca2LTR, cell-associated DNA (caDNA), cell-associated unspliced RNA (caRNA) from peripheral PBMCs, and plasma HIV-1 RNA using a low-copy assay [11, 12]. Although, the role of HIV-1 ca2LTR as a marker of residual replication remains debated [15, 16], evaluating this phenomenon in a well-controlled study using an improved INSTI remains valuable. Finally, although there was no impact of ART intensification on NCI, we also present exploratory analysis of whether virological markers are correlated with NCI severity, independent of ART intensification.

## METHODS

### Overall Trial Design

Those eligible for A5324 enrollment were PWH with plasma HIV-1 RNA levels <50 copies/mL on a stable ART regimen for ≥6 months that did not contain an INSTI or MVC and with NC performance ≥1 standard deviation below the normative mean on ≥2 NC tests in different domains. Of the 191 enrolled participants, 63 were randomized to dual PBO, 67 to DTG + PBO, and 61 to DTG + MVC. Further details regarding the design of the trial are available at clinicaltrials.gov (NCT02519777) and were published elsewhere [7]. The institutional review board at each site approved all study procedures and all participants provided written informed consent. Additional details regarding the NC assessment of participants are provided in the Supplementary Methods.

### Measurement of HIV-1 caDNA), HIV-1 caRNA,, HIV-1 ca2LTR, and Plasma Low-Level HIV-1 RNA

PBMCs and plasma were collected at before entry, at entry, and at weeks 2, 4, and 12. Total DNA was extracted from a frozen pellet of 4 × 10^6^ PBMCs for measurement of HIV-1 caDNA, HIV-1 ca2LTR, and ribonuclease P/MRP subunit p30 (RPP30) (for cell number normalization) by droplet digital polymerase chain reaction (ddPCR). The HIV-1 caDNA primer-probe targets a region in integrase and detects total HIV-1 DNA in cells, including intact provirus, defective provirus with an intact primer-probe site, and episomal HIV-1 DNA [17]. The HIV-1 ca2LTR assay primer–probe targets the LTR-LTR junction unique to 2-LTR circle products [17]. Both the HIV-1 caDNA and ca2LTR assay results were normalized to 1 × 10^6^ cell equivalents using the RPP30 copy number estimate determined by ddPCR. Total RNA was extracted from a frozen pellet of 1 × 10^6^ PBMCs for measurement of HIV-1 caRNA (fully unspliced HIV-1 RNA) using the same primer-probe set in the caDNA assay [17]. Total HIV-1 caRNA copies in the elute were calculated and assumed to be representative of the total cell number input into the extraction, in this case 1 × 10^6^ cells.

Based on a precision criterion of a standard deviation of base 10 log-transformed data ≤0.3 the lower limit of quantification (LLoQ) was 29, 7, and 60 copies/10^6^ PBMCs, respectively, for the HIV-1 caDNA assay, the HIV-1 ca2LTR assay, and the HIV-1 caRNA assay. Based on a 95% detection rate the limit of detection (LoD) of each assay matched the LLoQ. For plasma RNA, virus was pelleted from 6 mL of plasma, and HIV-1 RNA was measured using the Abbott RealTime HIV-1 Amplification Reagent Kit, with an LoD and LLoQ of 7 copies/mL. Further details regarding PBMC specimen preparation, HIV-1 cell-associated marker assays, low-copy assay to measure plasma HIV-1 RNA, and methods for measuring plasma concentrations of DTG and MVC are provided in the Supplementary Methods.

### Statistical Analysis

For all virological markers, undetectable results were set to 0. Detectable results less than the LLoQ were imputed to half the LLoQ. Pairwise comparisons for change over time were assessed using Wilcoxon rank sum tests for continuous measures and χ^2^ tests for categorical measures. All statistical tests were 2 sided and interpreted at a nominal 5% significance level without adjustment for multiple comparisons, consistent with the exploratory nature of the analyses. Additional details regarding the statistical analysis are provided in the Supplementary Methods.

## RESULTS

### Demographic and Disease Baseline Characteristics

Participant characteristics at baseline in the 3 treatment arms are shown in Table 1. Of the 191 enrolled participants, 171 are included in this analysis: 165 had all biomarker data available (56 dual PBO, 57 DTG + PBO, 52 DTG + MVC), 3 had cell-associated virological data but did not have plasma HIV-1 RNA data available at all time points (2 dual PBO, 0 DTG + PBO, 1 DTG + MVC), and 3 had plasma HIV-1 RNA data available at all time points but did not have cell-associated virological data at all time points (1 dual PBO, 0 DTG + PBO, 2 DTG + MVC). Excluded participants either went off study treatment before week 12 (4 participants; 1 dual PBO, 2 DTG + PBO, 1 DTG + MVC) or did not have data available at each time point. Included participants exhibited a range of NCI based on HAND diagnosis (37% with asymptomatic neurocognitive impairment, 53% with mild NC disorder, and 10% with HIV-associated dementia). Participant characteristics were reasonably balanced between the different treatment arms, including baseline levels for each of the virological markers examined in this study (Table 1).

**Table 1.** Participant Characteristics

Characteristic	Participants by Treatment Arm, No. (%)^a^			
	Dual PBO (n = 59)	DTG + PBO (n = 57)	DTG + MVC (n = 55)	Total (N = 171)
Age, mean (SD), y	52.0 (6.9)	52.0 (8.9)	51.9 (8.3)	52.0 (8.0)
Sex				
Male	46 (78)	36 (63)	37 (67)	119 (70)
Female	13 (22)	21 (37)	18 (33)	52 (30)
Race				
Asian	5 (8)	5 (9)	5 (9)	15 (9)
Black or African American	28 (47)	25 (44)	32 (58)	85 (50)
White	21 (36)	25 (44)	16 (29)	62 (36)
American Indian	1 (2)	0 (0)	0 (0)	1 (1)
>1 Race	1 (2)	1 (2)	1 (2)	3 (2)
Unknown	3 (5)	1 (2)	1 (2)	5 (3)
Ethnicity				
Hispanic or Latino	15 (25)	16 (28)	10 (18)	41 (24)
Not Hispanic or Latino	44 (75)	41 (72)	45 (82)	130 (76)
Previous IDU				
No	57 (97)	49 (86)	51 (93)	157 (92)
Yes	2 (3)	8 (14)	4 (7)	14 (8)
HIV-1 RNA <50 copies/mL^b^	58 (98)	55 (96)	53 (96)	166 (97)
HIV-1 markers, median (IQR)^c^				
caDNA	240 (149–384)	282 (134–530)	220 (93–437)	250 (128–477)
caRNA	51 (30–104)	60 (30–171)	30 (30–93)	53 (30–120)
ca2LTR	8.0 (0.0–21.7)	10.1 (0.0–18.7)	4.8 (0.0–14.5)	…
CD4 cell count, mean (SD), cells/µL	665 (282)	709 (275)	738 (334)	703 (298)
CD4/CD8 ratio, mean (SD)	0.9 (0.5)	1.0 (0.5)	1.1 (0.6)	1.0 (0.5)
Nadir CD4 cell count category^d^				
0–100/µL	18 (31)	16 (28)	16 (29)	50 (29)
>100/µL	41 (69)	41 (72)	39 (71)	121 (71)
Nadir CD4 cell count, median (IQR), cells/µL^e^	194 (91–372)	211 (65–338)	221 (74–354)	211 (74–354)
Total neuro *z* score, mean (SD)	−1.0 (0.8)	−1.0 (0.7)	−1.1 (0.7)	−1.0 (0.7)
BMI, mean (SD) µL^f^	29.5 (6.8)	29.0 (7.1)	29.1 (6.7)	29.2 (6.8)
BMI category ^f^				
Underweight (<18.5)	0 (0)	0 (0)	1 (2)	1 (1)
Normal (18.5–<25)	20 (34)	19 (33)	11 (20)	50 (29)
Overweight (25–<30)	17 (29)	21 (37)	23 (42)	61 (36)
Obese (≥30)	22 (37)	17 (30)	20 (36)	59 (35)
No. of comorbid conditions, mean (SD)	4 (4)	5 (5)	5 (5)	5 (5)
PI in regimen	20 (34)	21 (37)	21 (38)	62 (36)
Efavirenz use	24 (41)	17 (30)	17 (31)	58 (34)
Polypharmacy (>5 drugs)	20 (34)	22 (39)	22 (40)	64 (37)
HAND diagnosis				
ANI	21 (36)	21 (37)	21 (38)	63 (37)
MND	34 (58)	28 (49)	29 (53)	91 (53)
HAD	4 (7)	8 (14)	5 (9)	17 (10)

Abbreviations: ANI, asymptomatic neurocognitive impairment; BMI, body mass index; ca2LTR, cell-associated 2–long terminal repeat circles; caDNA, cell-associated DNA; caRNA, cell-associated unspliced RNA; DTG, dolutegravir; HAD, HIV-associated dementia; HAND, HIV-associated neurocognitive disorders; HIV, human immunodeficiency virus; IDU, intravenous drug use; MND, mild neurocognitive disorder; MVC, maraviroc; PBO, placebo; PI, protease inhibitor.

^a^Data represent no. (%) of participants unless otherwise specified.

^b^Although having an HIV-1 plasma RNA viral load <50 copies/mL was a requirement for eligibility, a small percentage of individuals had viral loads >50 copies/mL measured at baseline after enrollment.

^c^Median values are based on the numbers of participants with HIV-1 cell-associated marker data at baseline, which differed from the numbers in the column headings, as follows: dual-PBO arm, n = 58; DTG + PBO arm, n = 57; DTG + MVC arm, n = 53; and total group, n = 168.

^d^Previously documented nadir CD4 cell counts were used. If documented values were not available, self-reported values were used.

^e^Median nadir CD4 cell counts include only participants with previously documented nadir CD4 cell counts. Documented counts were available for 49 of 59 participants in the dual-PBO arm, 47 of 57 in DTG + PBO arm, 49 of 55 in the DTG + MVC arm, and 145 of 171 in the total group.

^f^BMI calculated as weight in kilograms divided by height in meters squared.

### Median DTG and MVC Plasma Concentrations measured at Week 12 and Week 48 Exceeded Expected Inhibitory Concentrations

The median plasma DTG trough concentration in the DTG/MVC arm was 887 ng/mL at week 12 and 877 ng/mL at week 48, exceeding the DTG in vitro 50% inhibitory concentration (IC_50_; non–protein binding adjusted) of 0.2 ng/mL against HIV-1 Ba-L (Supplementary Table 1) [10]. The median plasma MVC trough concentration in the DTG/MVC arm was 78 ng/mL at week 12 and 65 ng/mL at week 48, both of which were above the MVC in vitro IC_50_ of 0.26 ng/mL against CCR5-tropic HIV-1 [18]. The median plasma DTG trough concentration in the DTG/PBO arm was 903 ng/mL at week 12 and 905 ng/mL at week 48, also exceeding the in vitro IC_50_. The high median DTG and MVC trough concentrations between weeks 12 and 48 suggest steady-state pharmacokinetic conditions and good medication adherence.

### HIV-1 caDNA and caRNA Did Not Change Over the First Twelve Weeks of Intensification

**Figure 1.**
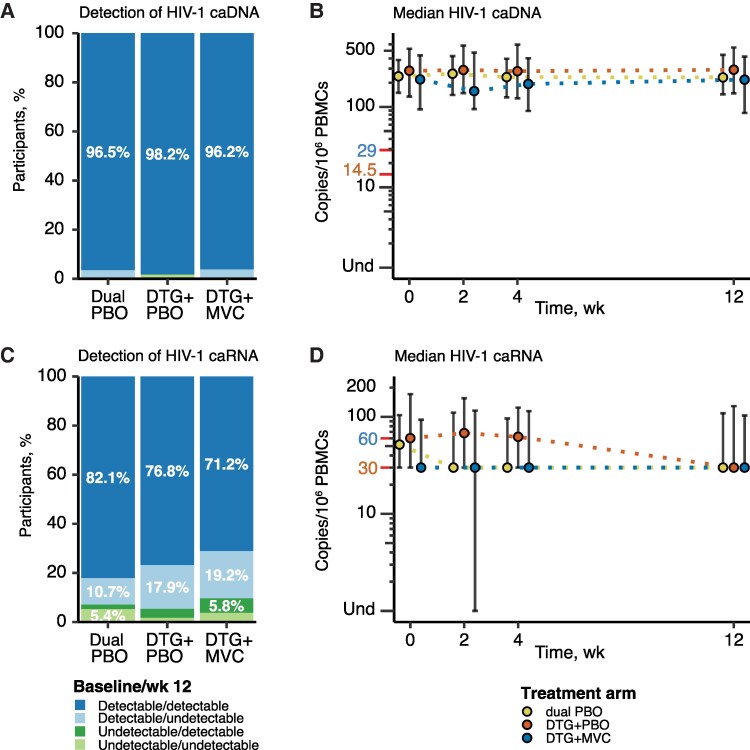
Detection and changes in median levels of human immunodeficiency virus (HIV) type 1 cell-associated DNA (caDNA) or cell-associated unspliced RNA (caRNA) between baseline and week 12. *A, C,* Results were categorized into 4 categories of detection patterns between baseline and week 12 for HIV-1 caDNA (*A*) and HIV-1 caRNA (*C*) . Detected results include those that were detected but fell below the limit of detection. The first 2 categories are detectable at baseline and either detectable (*dark blue*) or undetectable (*light blue*) at week 12; the second 2 are undetectable at baseline and either detectable (*dark green*) or undetectable (*light green*) at week 12. The proportions for each detection category are shown for each treatment arm (proportions <5% are not shown but are reported in Supplementary Table 10). A χ^2^ test was applied to determine whether there were significant differences in the pattern of detection; no significant differences were found (*P* > .05), and *P* values are not shown. *B, D,* Median values and interquartile range are plotted for HIV-1 caDNA (*B*) and HIV-1 caRNA (*D*) for each time point collected. Results from the dual-placebo (PBO) arm are plotted as yellow-filled circles, those from the dolutegravir (DTG) + PBO arm as orange-filled circles, and those from the DTG + maraviroc (MVC) arm as blue-filled circles. Undetectable values (Und) were assigned a value of 0, and the position is indicated on y-axis, which also displays the positions of half the lower limit of quantification (LLoQ) (*orange text and red tick mark*; 14.5 copies/10^6^ peripheral blood mononuclear cells [PBMCs] for HIV-1 caDNA and 30 copies/10^6^ PBMCs for HIV-1 caRNA) and the LLoQ (*blue text and red tick mark*; 29 copies/10^6^ PBMCs for HIV-1 caDNA and 60 copies/10^6^ PBMCs for HIV-1 caRNA).

Detection of HIV-1 caDNA between treatment arms did not differ substantively (dual PBO vs DTG + PBO, *P* = .22; dual PBO vs DTG + MVC, *P* = .94; DTG + PBS vs DTG + MVC, *P* = .21) (Figure 1*A*), and most participants had detectable HIV-1 caDNA at both baseline and week 12 (Figure 1*A* and Supplementary Figure 1*A*). Median HIV-1 caDNA levels were similar between treatment arms over time, but the DTG + MVC arm trended lower from baseline through the 12 weeks (Figure 1*B* and Supplementary Table 3). The median changes in HIV-1 caDNA levels between baseline and week 12 were similar across arms (Figure 1*B* and Supplementary Table 2). HIV-1 caRNA was less frequently detected, especially at both baseline and week 12, with only 82.1%, 76.8%, and 71.2% of participants having detectable caRNA at both time points in the dual-PBO, DTG + PBO, and DTG + MVC arms, respectively (Figure 1*C*). Changes in detection between baseline and week 12 did not differ between arms (dual PBO vs DTG + PBO, *P* = .49; dual PBO vs DTG + MVC, *P* = .39; DTG + PBO vs DTG + MVC, *P* = .84) (Figure 1*C*).

Like HIV-1 caDNA levels, HIV-1 caRNA median levels were similar between treatment arms over the 12 weeks of intensification (Figure 1D and Supplementary Table 4), with the DTG + MVC arm exhibiting the lowest level at each time point. Changes in median HIV-1 caRNA levels between baseline and week 12 were similar across arms (Figure 1D and Supplementary Table 2). Finally, we did not find differences in the HIV-1 caRNA/caDNA ratio between arms at baseline and week 12 or change in ratio from baseline to week 12 (Supplementary Table 5). In summary, we did not observe significant changes between arms in detection or copy levels of HIV-1 caDNA or HIV-1 caRNA over the first 12 weeks of intensification.

### HIV-1 ca2LTR Detectability Declined Over the First Twelve Weeks of Intensification

**Figure 2.**
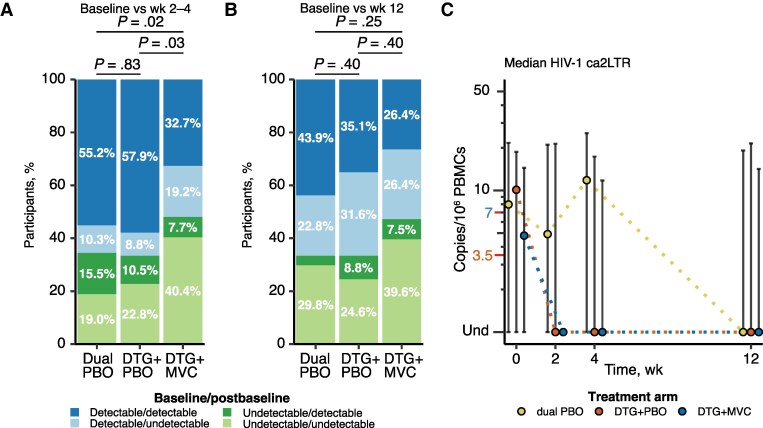
Detection and changes in median levels of human immunodeficiency virus (HIV) type 1 cell-associated 2–long terminal repeat circles (ca2LTR) between baseline and weeks 2–4 and week 12. *A, B,* Results were categorized into 4 categories of detectability patterns between baseline and weeks 2–4 (*A*) or week 12 (*B*). Detected results include those that were detected but fell below the limit of detection. The first 2 categories are detectable at baseline and either detectable (*dark blue*) or undetectable (*light blue*) after the baseline; the second 2 are undetectable at baseline and either detectable (*dark green*) or undetectable (*light green*) after the baseline. The proportions for each detection category are shown for each treatment arm (proportions <5% are not shown but are reported in Supplementary Table 10). A χ^2^ test was applied to determine whether there were significant differences in the pattern of detection between arms, and *P* values are displayed. *C,* Median values with interquartile range for HIV-1 ca2LTR are plotted for each time point collected. Results are plotted as yellow-filled circles for the dual–placebo (PBO) arm, as orange-filled circles for the dolutegravir (DTG) + PBO arm, and as blue-filled circles for the DTG + maraviroc (MVC) arm. Undetectable (Und) values were assigned a value of 0, and the position is indicated on the y-axis, which also displays the positions of half the lower limit of quantification (LLoQ) (*orange text and red tick mark*; 3.5 copies/10^6^ peripheral blood mononuclear cells [PBMCs]) and the LloQ (*blue text and red tick mark*; 7 copies/10^6^ PBMCs).

HIV-1 ca2LTR detection at weeks 2–4 was less frequent in the DTG + MVC arm than in the other arms (DTG + MVC, 40.4%; DTG + PBO, 68.4%; dual PBO, 70.7%; dual PBO vs DTG + MVC, *P* = .02; DTG + PBO vs DTG + MVC, *P* = .03 [based on χ^2^ tests] (Figure 2*A*). In contrast to the DTG + MVC arm, the pattern of ca2LTR detection between baseline and weeks 2–4 in the DTG + PBO arm did not differ from that in the dual-PBO arm (dual PBO vs DTG + PBO, *P* = .83) (Figure 2*A*). Overall, HIV-1 ca2LTR detection declined in all arms when comparing baseline to week 12, and the difference in HIV-1 ca2LTR detection in the DTG + MVC arm no longer held at week 12 (dual PBO vs DTG + PBO, *P* = .40; dual PBO vs DTG + MVC, *P* = .25; DTG + PBO vs DTG + MVC, *P* = .40) (Figure 2*B*). Similar to finding for HIV-1 caDNA and caRNA, the DTG + MVC arm generally exhibited the lowest median and mean concentrations of HIV-1 ca2LTR throughout the first 12 weeks of intensification (Figure 2*C* and Supplementary Table 6).

The trends in HIV-1 ca2LTR were less stable than for the other measures, with many participants fluctuating between detectable and undetectable independent of treatment arm (Supplementary Figure 2). Changes in median HIV-1 ca2LTR levels between baseline and week 12 were similar between treatment arms (Supplementary Table 2), and this levels trended higher in the dual-PBO arm at weeks 2 and 4 (Figure 2*C* and Supplementary Table 6). The distribution of participants experiencing an increase or decrease in HIV-1 ca2LTR differed only in the DTG + MVC arm, with a higher proportion categorized with a low, undeterminable change (baseline through week 4 measurements were all below either the LoD or the LLoQ) and a lower proportion categorized with an increase (Table 2 and Supplementary Figure 2). The subset of individuals receiving a protease inhibitor (PI) did not experience a transient increase in HIV-1 ca2LTR at weeks 2–4 (Supplementary Table 7). In summary, we observed changes in the detection of HIV-1 ca2LTR in the DTG + MVC arm early after intensification but no changes in the detectable HIV-1 ca2LTR levels or arm-specific transient increases in HIV-1 ca2LTR.

**Table 2.** Qualitative Changes in HIV-1 Cell-Associated 2–Long Terminal Repeat Circles Between Baseline and Weeks 2–4 by Treatment Arm

Treatment Arm	Change in HIV-1 ca2LTR, No. (%)			*P* Value		
	Increase	Decrease	Undetermined^a^	Versus Dual PBO	Versus DTG + PBO	Versus DTG + MVC
Dual PBO^b^	26 (45)	21 (36)	11 (19)	…	…	…
DTG + PBO	24 (42)	20 (35)	13 (23)	.88	…	…
DTG + MVC^b^	9 (17)	22 (42)	21 (40)	.004	.01	…

Abbreviations: ca2LTR, cell-associated 2–long terminal repeat circles; DTG, dolutegravir; HIV, human immunodeficiency virus; MVC, maraviroc; PBO, placebo.

^a^Results are undetermined if HIV-1 ca2LTR was undetectable or below the lower limit of quantification for all time points from baseline through week 4.

^b^One participant in the DTG + MVC arm had missing weeks 2 and 4 measures and was excluded from this analysis. One in the dual-PBO arm had weeks 2 and 4 measures, was missing week 12, and was included in this analysis.

### HIV-1 Plasma RNA Levels Were Not Affected by Twelve Weeks of Intensification

**Figure 3.**
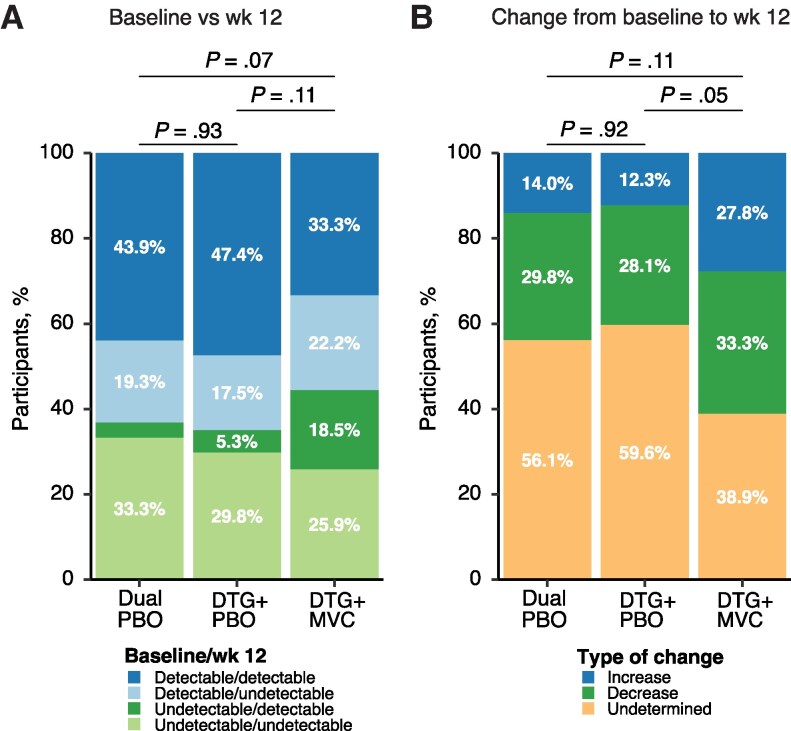
Detectability of plasma human immunodeficiency virus (HIV) type 1 RNA between baseline and week 12 and trends in plasma HIV-1 RNA between baseline and week 12. A, Results were categorized into 4 categories of detectability patterns between baseline and week 12. Detected results include those that were detected but fell below the limit of detection. The first 2 categories are detectable at baseline and either detectable (*dark blue*) or undetectable (*light blue*) at week 12; the second 2 categories are undetectable at baseline and either detectable (*dark green*) or undetectable (*light green*) at week 12. The proportions for each detectability category are shown for each treatment arm (proportions <5% are not shown but are reported in Supplementary Table 10). *B,* Results were categorized by whether there was an observed increase (*dark blue*), an observed decrease (*dark green*), or an undetermined change (*light orange*) between baseline and week 12. Detectable changes included results that went from undetectable to detectable (or vice versa), those that went from detectable below the lower limit of quantification (LloQ) to quantifiable (or vice versa), or quantifiable increases or decreases. Undetermined results were either undetectable or detected below the LloQ at both at both baseline and week 12. For both *A* and *B,* χ^2^ tests were applied to determine whether there were significant differences in the pattern of detectability between arms, and *P* values are displayed.

Detection of plasma HIV-1 RNA, as measured by a low-copy assay, did not differ between baseline and week 12 by treatment arm (Figure 3*A*), although the proportion of participants in the DTG + MVC arm in whom HIV-1 RNA was undetectable at baseline and then detectable at week 12 was 5.4-fold and 3.5-fold higher than in the dual-PBO and DTG + PBO arms, respectively. As expected for individuals on ART, the median viral load was less than the LLoQ (assigned at 3.5 copies/mL) or undetected (assigned at 0 copies/mL) (Supplementary Table 8). Finally, results were categorized by whether the change in viral loads between baseline and week 12 was an increase, a decrease, or undetermined (based on both values being below the LLoQ).

Analysis of these categories showed a modest difference between the DTG + PBO and the DTG + MVC arms but with not the other pairwise comparisons (dual PBO vs DTG + PBO, *P* = .92; dual PBO vs DTG + MVC, *P* = .11; DTG + PBO vs DTG + MVC, *P* = .05) (Figure 3*B*). The main difference was that the proportion of individuals with a detectable increase in plasma HIV-1 RNA between baseline and week 12 trended higher in the DTG + MVC arm (2.0- and 2.3-fold higher than in the dual-PBO and DTG + PBO arms, respectively) (Figure 3*B, blue shaded category*). Thus, HIV-1 plasma RNA levels appeared to increase in a greater proportion of participants in the DTG + MVC arm, in contrast to the results with HIV-1 ca2LTR marker.

HIV-1 virological markers were not correlated with NC performance average *z* scores or change in average *z* scores between baseline and week 48, and no specific baseline characteristic was associated with individuals who experienced a decline in any of the HIV-1 virological markers.

Since we did not observe changes in median levels of HIV-1 caDNA, caRNA, ca2LTR, and plasma RNA (Figure 1*B* and 1*D*, Figure 2C, and Supplementary Tables 2–4 and 6) between treatment arms and mean average NC performance *z* scores were similar between arms at baseline (Table 1) and over the course of the study [7], we next considered whether there was any correlations between each viral marker and NC performance across the entire cohort. There were no strong correlations between these markers and average NC performance *z* score at baseline (*P* > 0.05) (Table 3), though an increase in HIV-1 ca2LTR levels between baseline and week 12 was weakly associated with improvement in average NC performance *z* score between baseline and week 48 (*r* = 0.15; *P* = .05) (Table 3). Higher ca2LTR levels at baseline were associated with better fine motor skills (*r* = 0.16; *P* = .04), and increases in ca2LTR were associated with improvements in several cognitive domains (executive function, *r* = 0.19 and *P* = .01; fine motor skills, *r* = 0.22 and *P* = .004; attention/working memory, *r* = 0.19 and *P* = .01) (Supplementary Table 9).

**Table 3.** Correlation Between Each HIV-1 Marker and Total *z* Score at Baseline and Between Change From Baseline to Week 12 in Each Marker With Change From Baseline to Week 48 in Total *z* Score

HIV-1 Marker	Participants, No.	Correlation With Total *z* Score or Change in Total *z* Score	*P* Value
Baseline			
caDNA	168	−0.02	.79
caRNA	168	−0.07	.34
ca2LTR	168	0.01	.91
Plasma RNA	168	−0.02	.75
Change from baseline			
caDNA	167	0.02	.81
caRNA	164	−0.11	.16
ca2LTR	167	0.15	.05
Plasma RNA	168	0.07	.36

Abbreviations: ca2LTR, cell-associated 2–long terminal repeat circles; caDNA, cell-associated DNA; caRNA, cell-associated unspliced RNA; HIV, human immunodeficiency virus.

Next, to determine whether any of the baseline characteristics were associated with a decline in any of the HIV-1 markers, the entire cohort was divided as follows. First, we compared the group in which individuals had detectable values at baseline and week 12 versus the group in which values were detectable at baseline but undetectable at week 12 (loss of detection). Second, we included in the loss-of-detection group those whose values showed a quantitative decline but were still detectable (loss of detection or decline). For the HIV-1 caDNA assay no single baseline characteristic differed by either criterion (Supplementary Table 11). For the HIV-1 caRNA assay, sex and race differed in the loss-of-detection comparison, with undetectable values at week 12 in a lower proportion of male participants (72% in those still detectable at week 12 vs 54% in those no longer detectable at week 12), a higher proportion of individuals who identify as Asian (6% vs 23%), and a lower proportion of those who identify as white (40% vs 19%). For the HIV-1 ca2LTR assay, intravenous drug history was associated with either comparison, but the distributions of individuals without or with intravenous drug use history were not consistent between comparisons. Finally, for the low-level HIV-1 RNA from plasma assay, the average age was lower in the group with undetectable values at week 12 (50.7 vs 54.0 years; *P* = 0.06), and lower body mass index (BMI) was associated with the group had undetectable values at week 12 or experienced a quantitative decline (BMI, 27.5 vs 29.9; *P* = .07). In summary, no single baseline characteristic was associated with decline in all HIV-1 markers.

## DISCUSSION

The A5324 trial was the largest randomized controlled trial of ART intensification in PWH with NCI to date. To determine whether ART intensification affected residual replication, HIV-1 caDNA, caRNA, ca2LTR, and plasma RNA were monitored from the periphery over the first 12 weeks. Based on this testing, we found that intensification with DTG + PBO or DTG + MVC had no significant impact on peripheral markers of HIV-1 persistence or peripheral residual replication.

Past studies of ART intensification in PWH with MVC [19–22] or INSTIs [11, 13, 14, 23–27] did not observe effects on HIV-1 caDNA, caRNA, or plasma RNA levels. Significant decreases in HIV-1 caRNA were observed in 1 study of MVC + raltegravir [28] while another study of MVC intensification found HIV-1 caRNA trended higher, possibly due to increasing HIV-1 transcription via NFκB [29, 30]. In agreement with most studies of intensification, we did not observe meaningful differences between treatment arms in HIV-1 caDNA or caRNA (Figure 1). We did observe a modestly higher proportion of individuals experiencing an increase in HIV-1 plasma RNA in the DTG + MVC arm compared with the DTG + PBO arm (Figure 3). One possible explanation for this increase could be explained by MVC increasing HIV-1 transcription and virus production; however, such an effect was not observed in HIV-1 caRNA measurements. In addition, the relative change in HIV-1 plasma RNA was small, as most of these participants remained below the LLoQ of standard clinical assays.

In contrast to other HIV-1 virological markers, the effects of ART intensification on HIV-1 ca2LTR levels have been more mixed. Transient increases in ca2LTR have been observed in some studies of raltegravir intensification [11, 12, 26] but not in others [13, 14, 24]. Interestingly, transient increases in ca2LTR were most prominent in PWH receiving ART that included a PI [11, 12, 26], suggesting that viral replication may be incompletely suppressed. Consistent with this speculation, raltegravir intensification has been associated with a decrease in HIV-1 caRNA when combined with ART regimens that included a PI [25, 28]. This phenomenon is not universal to INSTIs since transient increases in HIV-1 ca2LTR were not observed with DTG intensification, even in the subset of participants with a PI included in their ART regimen [27].

The effect of MVC intensification on HIV-1 ca2LTR is also mixed, with 1 study finding a transient increase after 24 weeks of intensification in a small number of individuals [19] and other studies finding no significant changes [21] including 1 intensification that included both MVC and raltegravir [28]. We also did not observe an early transient increase in HIV-1 ca2LTR, even within the subset also receiving a PI (Table 2 and Supplementary Table 7) [27]. One possible explanation for the different findings between INSTI regimens is that raltegravir can achieve higher concentrations in the gut relative to plasma compared with DTG (600-fold vs 0.83-fold, respectively) and may be more effective at inhibiting ongoing replication in the gut tissue site [31, 32]. Unexpectedly, we saw lower HIV-1 ca2LTR detection in the DTG + MVC arm than in the other treatment arms in weeks 2–4, but this difference was diminished by week 12 (Figure 2*A* and 2*B*), perhaps due to improved drug adherence across arms. Baseline caDNA and caRNA levels were also lower in the DTG + MVC arm, so it is unclear whether the lowered ca2LTR detection in the DTG + MVC arm was a consequence of intensification or a failure in randomization (Supplementary Tables 3 and 4).

We did not observe any strong correlations at baseline or change over time between HIV-1 virological markers and average NC *z* score or individual cognitive domain *z* scores (Table 3 and Supplementary Table 9). Some studies have shown a correlation between HIV-1 caDNA and the occurrence of HIV-associated dementia or performance on NC tests [33]. Others have found a correlation between NC performance and detectable plasma HIV-1 RNA [34] or levels of HIV-1 RNA in cerebrospinal fluid [35, 36]. This study included individuals experiencing the less severe forms of HAND (asymptomatic neurocognitive impairment and mild NC disorder), where a correlation between NC performance and peripheral virological markers may not be strong. ART in this cohort was already highly effective, since 1 criterion for eligibility included plasma HIV-1 RNA levels <50 copies/mL and the observed average *z* score might be associated with a CNS injury that predated the study or determinants that are independent of HIV-1.

There are several limitations to this study. All of the virological markers we measured were from peripheral blood, which may miss important changes occurring at tissue sites were HIV-1 is known to replicate or from cerebrospinal fluid where virological markers may better correspond with NCI [35–37]. The assays used in this analysis were rigorously validated and have sensitivity comparable to that of other published assays similarly validated [17], but analysis of CD4^+^ T cells instead of PBMCs may have improved our sensitivity by approximately 3-fold, allowing for better detection of subtle changes in these cell-associated HIV-1 markers. In addition, there are slightly more sensitive methods for low-copy plasma HIV-1 RNA (approximately 2–3 fold more sensitive) [38]. The HIV-1 caDNA assay used did not distinguish between defective and intact provirus, so specific effects on the intact reservoir may have been diluted by the much larger defective reservoir [39]. MVC may alter T-cell trafficking between the tissue sites and the periphery via CCR5 inhibition, possibly confounding some of the observations from the periphery [20]. This was an exploratory analysis, and the *P* values were not corrected for multiple comparisons, so some statistically significant findings may be spurious. Finally, we sought to assess possible effects on residual replication by monitoring HIV-1 ca2LTR, but the role of this marker is controversial, with more recent studies finding that it has a longer half-life, which may preclude it as a marker of recent replication events [15, 16].

Finally, 3 individuals experienced high plasma HIV-1 RNA levels (>1000 copies/mL) at ≥1 time point, and 4 individuals experienced transient increases in plasma HIV-1 RNA (50–200 copies/mL), which were included in the analysis (see Supplementary Methods, low-level plasma HIV-1 RNA, for additional details). When these individuals were excluded from the analysis there were small shifts in *P* values, but the overall interpretation remained the same (data not shown).

In summary, intensification with DTG or DTG + MVC did not affect either peripheral HIV-1 cell-associated markers or plasma HIV-1 RNA concentrations, indicating that intensification may not have affected residual replication. We cannot rule out effects specifically within the CNS that may not be reflected in measurements from the periphery; however, this study is consistent with other intensification studies demonstrating little impact on HIV-1 reservoir or residual replication. Alternative approaches are likely required to reduce the size of the reservoir and improve NC performance in PWH.

## Supplementary Material

ofaf594_Supplementary_Data

## Supplementary Data

Supplementary materials are available at *Open Forum Infectious Diseases* online. Consisting of data provided by the authors to benefit the reader, the posted materials are not copyedited and are the sole responsibility of the authors, so questions or comments should be addressed to the corresponding author.
