# Supplementary material for: Antiretroviral Therapy Intensification With Dolutegravir and/or Maraviroc Did Not Affect HIV-1 Cell-Associated DNA, RNA, and 2­–LTR Circles Over 12 Weeks
Source: Open Forum Infect Dis. 2025 Oct 1;12(10):ofaf594. doi: 10.1093/ofid/ofaf594 (PMC12548372; doi:10.1093/ofid/ofaf594)
Supplement: ofaf594_Supplementary_Data [file ofaf594_supplementary_data.zip › A5324_supplementary_Methods_v10.docx]

**Supplemental Methods**

**Preparation of peripheral blood mononuclear cells (PBMC) for extraction:** For virological marker assessments, blood was collected at pre-entry, entry, week 2, week 4, and week 12. Pre-entry was a median (IQR) of 7 (5-13) days prior to entry. PBMC were isolated at each local site following ACTG-approved procedures and stored at -80°C prior to aliquoting. In preparation for analysis, PBMC were viably thawed, resuspended in 1xHBSS supplemented with 5.6 mM glucose (ThermoFisher, 14175103) and aliquots of 4x10^6^ PBMC per vial or 1x10^6^ PBMC per vial were prepared for total DNA or total RNA extraction, respectively. Aliquots were stored a -80°C prior to extraction.

**Measurement of cell-associated HIV-1 DNA (caDNA), HIV-1 unspliced RNA (caRNA), HIV-1 2-LTR circle (ca2LTR):** Extractions and droplet digital PCR (ddPCR) assays for HIV-1 caDNA, HIV-1 caRNA, and HIV-1 ca2LTR were performed as previously described [1]. Briefly, total DNA to measure HIV-1 caDNA, HIV-1 ca2LTR, and ribonuclease P/MRP subunit p30 (RPP30) (for cell number normalization) was extracted from frozen 4x10^6^ PBMC cell pellets using a QIAamp DNA mini kit (Qiagen cat# 51306), with minor modifications to the manufacturer’s instructions [1], and DNA was eluted into 120 µL with 10 mM Tris-Cl pH 8.5 elution buffer (Qiagen # 19086). Each extraction includes several controls: a HIV-1 negative PBMC pellet, a PBMC pellet with a low amount of HIV-1 DNA or 2-LTR circle (200 copies of HIV-1 DNA, 100 copies 2-LTR circle), and a PBMC pellet with high amount of HIV-1 DNA or 2-LTR circle (20,000 copies of HIV-1 DNA, 1,000 copies 2-LTR circle). Primers and probes for the HIV-1 caDNA and HIV-1 ca2LTR ddPCR assays were as follows, HIV-1 DNA assay: forward 5´-GGTTTATTACAGGGACAGCAGA-3´; reverse 5´-CACACAATCATCACCTGCCA-3´; probe 5´-/56-FAM/TGTATTACT/ZEN/ACTGCCCCTTCACCTTTCCAGAG/3IABkFQ/-3´. 2-LTR assay: forward 5´- CAATAAAGCTTGCCTTGAGTGC -3´, reverse 5´- GTGTAGTTYTGCCAATCAGGG -3´, probe 5´-/56-FAM/ACTCTGGTA/ZEN/ACTAGAGATCCCTCAGACC/3IABkFQ/-3´. The RPP30 assay primers and FAM probe were purchased as a 20x stock that is ready-to-use (Biorad, Assay ID: dHsaCP1000485 targeting hg19|chr10:92634423-92634545). The ddPCR reactions were setup with 2x ddPCR Supermix probes no dUTP kit (Biorad, 1863024) using 9.75 µL of extracted DNA for HIV-1 caDNA and HIV-1 ca2LTR and 2 µL of extracted DNA for RPP30. Thermocycling conditions were as follows, 1 cycle at 95 °C for 10 minutes, 50 cycles at 94 °C for 30 seconds followed by 58 °C for 2 minutes, ending with 1 cycle at 98 °C for 10 minutes. For the HIV-1 DNA and 2-LTR assays, results were normalized to 1x10^6^ cell equivalents using the RPP30 copy number estimate determined by ddPCR. Total RNA to measure HIV-1 caRNA and TATA-Box Binding Protein (TBP) (positive control) was extracted from frozen 1x10^6^ PBMC cell pellets using the MagMax mirVana Total RNA kit (ThermoFisher, A27828), with minor modification to the manufacturer’s instructions, and RNA was eluted into a 100 µL volume. Each extraction includes several controls: a HIV-1 negative PBMC pellet, a PBMC pellet with low amount of HIV-1 usRNA (200 copies of HIV-1 usRNA), and a PBMC pellet with high amount of HIV-1 usRNA (20,000 copies of HIV-1 usRNA). The primer and probe used for RT-ddPCR for HIV-1 usRNA is the same as described for the HIV-1 caDNA ddPCR assay. The TBP assay primers and FAM probe were purchased as a 20x stock that is ready-to-use (Biorad, Assay ID: dHsaCPE5058362 targeting hg19|chr6:170871320-170876038 and spans over an exon-exon boundary). The RT-ddPCR reactions were setup with One-Step RT-ddPCR Advanced Kit for Probes (Bio-Rad, 1864021) using 10 µL of extracted RNA for HIV-1 caRNA and 1 µL of extracted RNA for TBP. Thermocycling conditions were as follows, 1 cycle at 50 °C for 60 minutes for reverse transcription, 1 cycle at 95 °C for 10 minutes, 50 cycles of 95 °C for 30 seconds followed by 58 °C for 2 minutes, ending with 1 cycle of 98 °C for 10 minutes. For the HIV-1 usRNA assay, total HIV-1 usRNA copies in the elute was calculated and assumed to be representative of the total cell number input into the extraction, in this case 1x10^6^ cells. The results from the low and high controls in all assays were tracked in a Levey-Jennings plot to monitor assay performance over time. Note these values differ from our publication of the same assays [1], which utilized a more stringent precision criteria of an arithmetic CV% ≤ 30% where the LLoQ of the HIV-1 caDNA assay is 70 copies/million PBMC, HIV-1 ca2LTR assay is 24 copies/million PBMC, and HIV-1 caRNA assay is 149 copies/million PBMC and the LoD values remain the same as described above. The estimated CV% at the LLoQ utilized for these ddPCR assays in the A5324 trial are 53.6%, 41.2%, and 48.6% for the HIV-1 caDNA, ca2LTR, and caRNA assays, respectively.

**Measurement of low-level HIV-1 RNA from plasma:** Subject plasma was thawed at room temperature and mixed well by vortexing. Six milliliters of plasma was split among four 2 mL microcentrifuge tubes (Sarstedt). Next, 400 µL Basematrix (SeraCare, 18050075) was added to the plasma specimen vial (or split evenly if more than one specimen vial), vortexed and spun to settle contents, and 100 µL of rinse was combined with each of the four plasma aliquots prepared above. Alongside the subject specimens, the following controls were prepared: negative control (Basematrix alone); VQA25 (25 copies/mL) prepared by the Viral Quality Assurance (VQA) program at Duke university; VQA5 (5 copies/mL) prepared internally by dilution of VQA200 (200 copies/mL) with Basematrix; CP20 (20 copies/mL) prepared internally by dilution of a previously tested HIV-1 RNA specimen with Basematrix. Virion material from the plasma was then pelleted by centrifugation at 30,000g at 4°C for 2 hours. Following centrifugation, any accumulated lipid on the top of the plasma was first removed prior to removing the plasma supernatant. The virion pellet was lysed by addition of 150 µL NucliSENS (bioMérieux, 280134), pulse vortexed for 15 seconds, followed by centrifugation at 14,000g for 3 minutes. Lysed material from four tubes were pooled into Simport tubes (Simport Scientific, T5014T) suitable for the *m*2000 and volumes normalized to 800 µL with the NucliSENS. Specimens were then extracted using Abbot *m*2000*sp* instrument and HIV-1 RNA measured using the Abbot RealTime HIV-1 Amplification Reagent Kit, following the manufacturer’s instructions. This assay was validated as follows. The LoD was established using probit analysis of results from testing of control material, provided by the Duke Human Vaccine Institute Virology Quality Assurance (VQA) program, diluted to concentrations of 2, 5, 8, and 12 HIV-1 RNA copies/mL. Based on this analysis, the LoD was determined to be 7 copies/mL, using a 6 mL input volume. Intra-assay and inter-assay imprecision were assessed through repeated testing of VQA positive control dilutions and clinical specimens with known HIV-1 RNA concentrations. The assay demonstrated acceptable precision, with a geometric standard deviation ≤ 2. Linearity was based on the established linearity of the Abbot RealTime HIV-1 assay utilized for this procedure with verification of linearity between 2-12 HIV-1 RNA copies/mL. Accuracy was evaluated by testing of high-copy samples (200–500 copies/mL) diluted 10-fold using Basematrix, resulting in expected final concentrations of 20–50 copies/mL. The assay showed acceptable accuracy, with recovered values within 0.7 log₁₀ HIV-1 RNA copies/mL of expected concentrations. Specificity was confirmed by testing both positive specimens (VQA standards and low-level clinical pools) and negative specimens (negative donor plasma or Basematrix) and the assay demonstrated 100% diagnostic specificity. Analytical specificity was assessed using a panel of contrived specimens containing common serum inhibitors (AcroMetrix Inhibition Panel, Thermo Scientific, 956400). Inhibition was observed in specimens with moderate to high hemolysis. As a result, severely hemolytic samples are excluded from low-level HIV-1 RNA testing. Finally, based on this validation the analytical measurement range of the assay is from 7 to 1.3x10^6^ HIV-1 RNA copies per mL. Three participants experienced higher than expected HIV-1 plasma RNA. One participant in the DTG+PBO arm had an entry level of >100,000 copies/mL and a week 2 level of >100 copies/mL. Lastly, two participants in the Dual PBO arm had week 12 level of >1000 copies/mL. Additionally, four participants experience a transient increase in plasma HIV-1 RNA at a single timepoint in the range of 50-200 copies/mL. All seven participants were included in the analysis of cell-associated HIV-1 virological markers and HIV-1 plasma RNA.

**Measurements of Dolutegravir (DTG) and Maraviroc (MVC) plasma levels:** DTG and MVC plasma concentrations were measured using validated analytical methods based on protein precipitation or solid phase extraction followed by high-performance liquid chromatography–tandem mass spectrometry [2,3]. The lower limit of quantification was 5 ng/mL (DTG) and 0.5 ng/mL (MVC), and the upper limit was 10,000 ng/mL (DTG) and 1,000 ng/mL (MVC). Plasma samples for trough concentration measurement were collected at 12 weeks and 48 weeks pre-dose.

**Neurocognitive Assessment:** Details regarding NC test battery, implementation of testing, and scoring were published previously [4]. Briefly, tests included measures of attention/working memory, speed of information processing, executive function, verbal learning, verbal memory, and fine motor skills. Raw scores were converted to *z* scores using country and site-specific norms, adjusting for age, sex, and educational attainment. The total *z* score was determined by taking the average of all the domain-specific *z* scores.

**Statistical Analysis:** Participants were included in the analysis if they had data available at both baseline (pre-entry or entry) and post-baseline (week 2, week 4, or week 12). The baseline estimate was set to either the pre-entry or entry values if only one was available or the average of pre-entry and entry measurements if both were available. For the HIV-1 ca2LTR analysis of week 2-4 data, the week 2 and 4 results were either averaged or if only one result was available that result was utilized. Change from baseline was evaluated categorically in two ways: (1) detectable/undetectable status at the two timepoints, resulting in 4 categories; (2) increase from baseline, decrease from baseline, or undetermined change, resulting in 3 categories. When evaluating an increase or decrease from baseline, changes included results that went from undetectable to detectable (or vice versa), detectable below the LLoQ to quantifiable (or vice versa), or a quantifiable increase or decrease. Pairwise comparisons for change over time were assessed using Wilcoxon rank-sum tests for continuous measures and Chi-square tests for categorical measures. Associations between virological markers and neurocognitive performance were analyzed using Spearman correlations. When evaluating the correlation in change of virological makers with change in neurocognitive performance over the study we calculated the change by subtraction of the post-baseline values (week 12 for virological markers and week 48 for neurocognitive performance) from the baseline values. Correlations were examined between each virological marker and total z score (**Table 3**) and HIV-1 ca2LTR and domain-specific *z* scores (**Table S9**). This was an exploratory analysis, and therefore, no formal sample size or power calculations were conducted prior to study initiation to estimate the effect sizes required to detect differences in virological markers. Accordingly, the results should be interpreted as hypothesis-generating. Nonetheless, based on the final group sizes across the different HIV-1 markers, we estimate that the study had approximately 80% power to detect a minimum between-group mean difference of 0.52 to 0.55 standard deviations, assuming approximate normality and a two-sided two-sample t-test with a Type I error rate of 0.05. Approximate detectable effect sizes were ~38 copies/million PBMC for the HIV-1 caDNA assay, ~22 copies/million PBMC for the HIV-1 caRNA assay, ~3 copies/million PBMC for the HIV-1 ca2LTR assay, and ~1.5 copies/mL for the low-level HIV-1 RNA from plasma assay. Analyses were performed using SAS version 9.4 (SAS Institute Inc.).

**References**

1. Reed J, Kwak G, Piliper EA, Degli-Angeli EJ, Goecker EA, Greninger AL. Validation of digital droplet PCR assays for cell-associated HIV-1 DNA, HIV-1 2-LTR circle, and HIV-1 unspliced RNA for clinical studies in HIV-1 cure research. Journal of Clinical Virology **2024**; 170:105632.

2. Bennetto-Hood C, Tabolt G, Savina P, Acosta EP. A sensitive HPLC–MS/MS method for the determination of dolutegravir in human plasma. Journal of Chromatography B **2014**; 945:225–232.

3. Fayet A, Béguin A, Zanolari B, et al. A LC–tandem MS assay for the simultaneous measurement of new antiretroviral agents: Raltegravir, maraviroc, darunavir, and etravirine. Journal of Chromatography B **2009**; 877:1057–1069.

4. Letendre SL, Chen H, McKhann A, et al. Antiretroviral Therapy Intensification for Neurocognitive Impairment in Human Immunodeficiency Virus. Clinical Infectious Diseases **2023**; 77:866–874.
