# Supplementary material for: Antiretroviral Therapy Intensification With Dolutegravir and/or Maraviroc Did Not Affect HIV-1 Cell-Associated DNA, RNA, and 2­–LTR Circles Over 12 Weeks
Source: Open Forum Infect Dis. 2025 Oct 1;12(10):ofaf594. doi: 10.1093/ofid/ofaf594 (PMC12548372; doi:10.1093/ofid/ofaf594)
Supplement: ofaf594_Supplementary_Data [file ofaf594_supplementary_data.zip › Fig_S1.pdf]

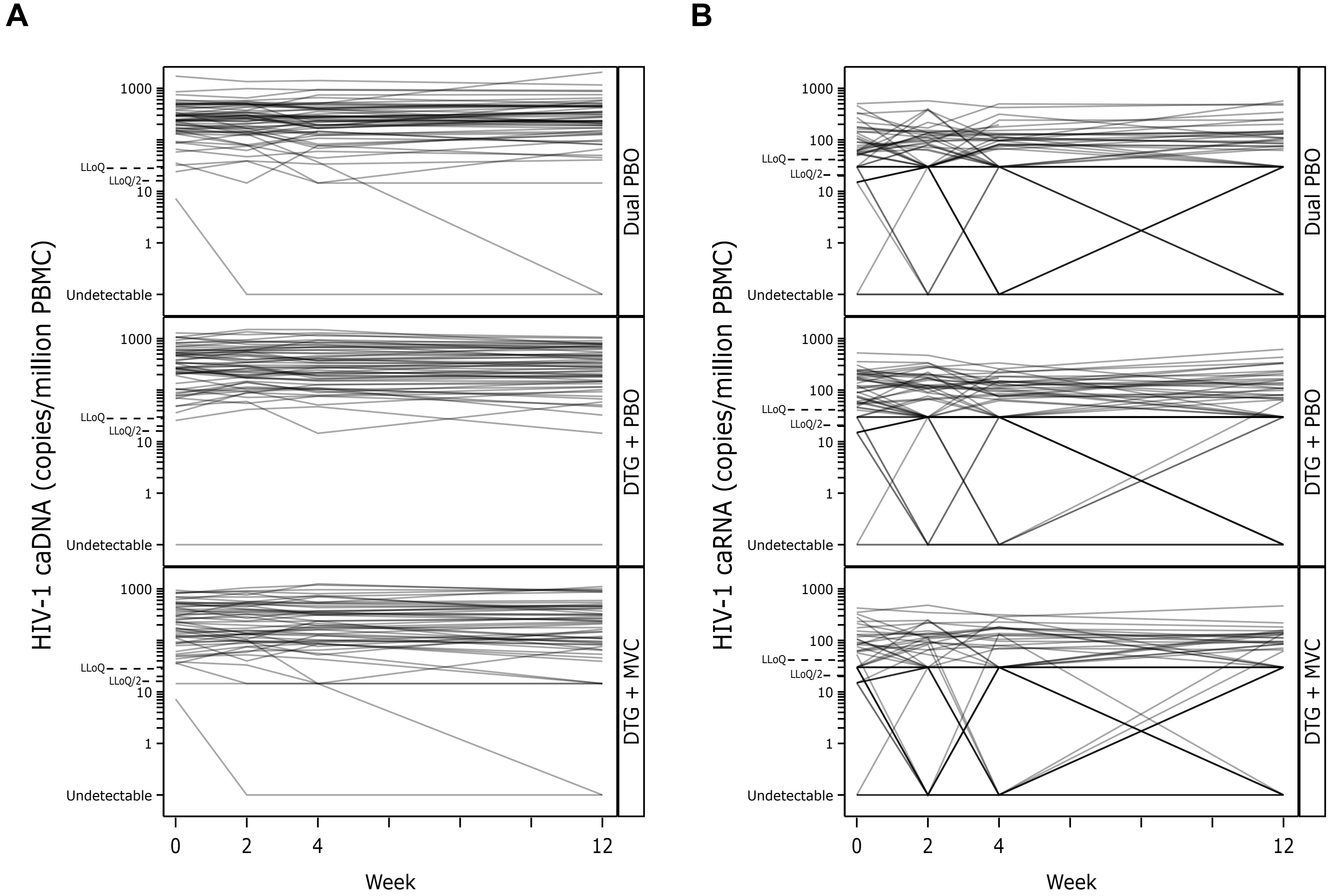

Supplementary Figure 1: Trends in HIV-1 caDNA or HIV-1 caRNA over the first twelve weeks of intensification. Plotted are individual trend lines over the first twelve weeks of intensification for (A) HIV-1 caDNA and (B) HIV-1 caRNA. When a result was undetectable for a timepoint it was plotted as zero and that position is marked on the y-axis. Detectable results less than the LLoQ were plotted at one-half LLoQ and the LLoQ and one-half LLoQ positions are marked on the y-axis.
